# Pro-inflammatory monocytes in patients with calcific aortic valve disease

**DOI:** 10.1093/cvr/cvaf148

**Published:** 2025-08-26

**Authors:** Wieteke Broeders, Amber van Broekhoven, Aysun Cetinyurek-Yavuz, Erwin Zegers, Anthonie L Duijnhouwer, Mihai G Netea, Siroon Bekkering, Niels van Royen, Saloua El Messaoudi, Niels P Riksen

**Affiliations:** Department of Internal Medicine, Radboud University Medical Center, Geert Grooteplein Zuid 10, Nijmegen 6525 GA, The Netherlands; Department of Cardiology, Radboud University Medical Center, Nijmegen, The Netherlands; Department of Health Evidence, Radboud University Medical Center, Nijmegen, The Netherlands; Department of Cardiology, Canisius Wilhelmina Hospital, Nijmegen, The Netherlands; Department of Cardiology, Radboud University Medical Center, Nijmegen, The Netherlands; Department of Internal Medicine, Radboud University Medical Center, Geert Grooteplein Zuid 10, Nijmegen 6525 GA, The Netherlands; Department of Internal Medicine, Radboud University Medical Center, Geert Grooteplein Zuid 10, Nijmegen 6525 GA, The Netherlands; Department of Cardiology, Radboud University Medical Center, Nijmegen, The Netherlands; Department of Cardiology, Radboud University Medical Center, Nijmegen, The Netherlands; Department of Internal Medicine, Radboud University Medical Center, Geert Grooteplein Zuid 10, Nijmegen 6525 GA, The Netherlands

**Time of primary review: 33 days**

There is a pressing need to elucidate the pathophysiology of calcific aortic valve disease (CAVD), because its prevalence and associated health burden is increasing and there is no pharmacological treatment option. Its risk factors and pathology show considerable overlap with atherosclerosis, in which inflammation and innate immune cells are key.^1^ In patients with coronary artery disease, circulating monocytes show a hyperinflammatory phenotype.^2^ Previous studies on monocytes in CAVD only focused on monocyte numbers and subsets,^3^ which is insufficient to unveil new treatment targets.

We now hypothesized that monocyte activation also contributes to CAVD. We tested this in 119 patients with a tricuspid aortic valve stenosis (TAVS) and 65 healthy controls (HC) (*Figure 1A*). Forty-eight patients had a mild/moderate AVS (M/M-TAVS), and 71 had severe AVS (S-TAVS). Patients were recruited at Radboud University Medical Center and Canisius Wilhelmina Hospital, after approval by the Medical Ethics Committee Region Arnhem-Nijmegen (NL72973.091.20). TAVS was defined by transthoracic echocardiography according to the 2017 ESC/EACTS guidelines.^4^ We excluded patients with anti-inflammatory drugs, auto-immune disease, infection, malignancy, and recent ischaemic events. Continuous baseline characteristics are compared using the one-way ANOVA (for normally distributed values with homogeneity of variances) or with the Kruskal–Wallis test. Categorical baseline variables are compared with the *χ*² test or Fisher’s exact test. Immunological variables are compared using univariate linear regression or logistic regression, with correction for age, sex, body mass index (BMI), smoking, systolic blood pressure, glucose, LDL-cholesterol, and season and year of blood collection, applying manual backward covariate selection. Correlations were calculated with the non-parametric Spearman correlation. For final significance, correction for multiple testing was performed for all analyses made per type of parameter group using the Benjamini–Hochberg method with a false discovery rate of 10%.

**Figure 1:**
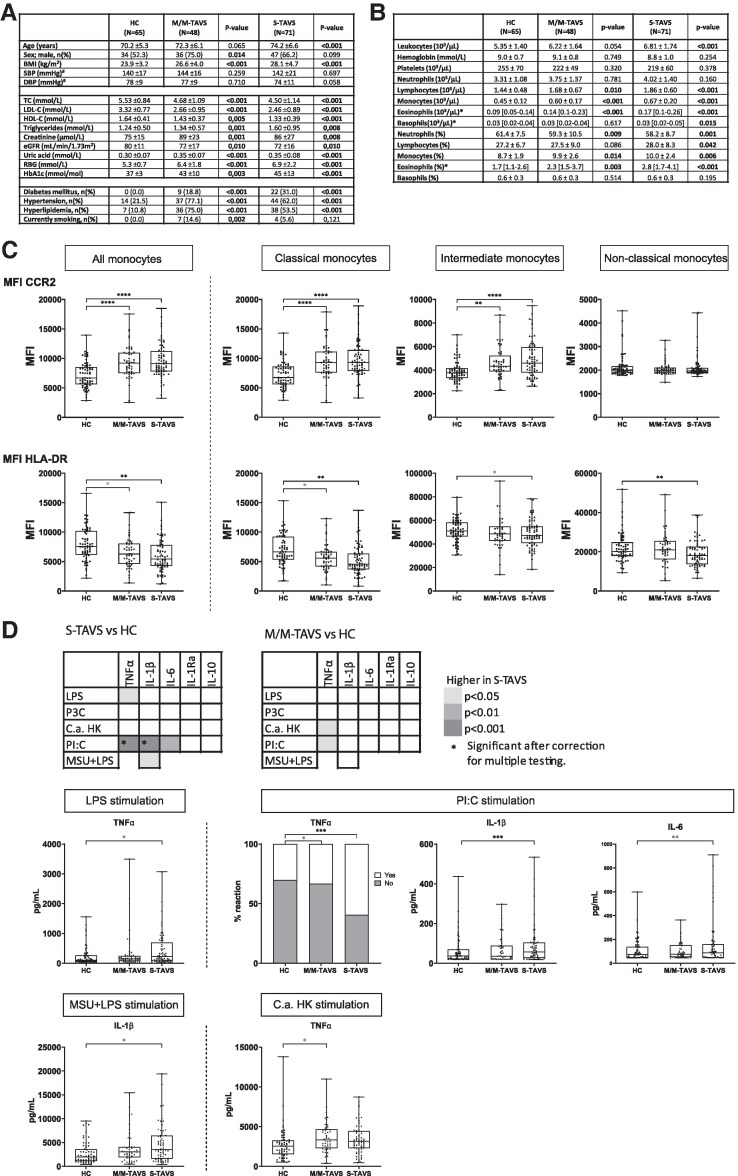
(*A*) Baseline characteristics. All parameters are expressed as mean ± SD or number (%) and compared to HCs. *P*-values that remained significant after correction for multiple testing (Benjamini–Hochberg method with a 10% false discovery rate) are given in bold. ^#^Data are missing for one HC. (*B*) Whole blood composition. Expressed as mean ± SD or median with interquartile range [Q1–Q3]. Values that remained significant after correction for multiples testing are given in bold. *Variables are log10 transformed for linear regression. (*C*) Flow cytometry results. The boxplots depict the MFI of CCR2 and HLA-DR on all monocytes and on the separate subsets. MFI CCR2: HC *n* = 65, M/M-TAVS *n* = 48, S-TAVS = 71. MFI CCR2 on non-classical monocytes: HC *n* = 65, M/M-TAVS *n* = 47, S-TAVS *n* = 71. MFI HLA-DR: HC *n* = 65, M/M-TAVS *n* = 47, S-TAVS *n* = 71. (*D*) PBMC cytokine production capacity. The heatmaps depict the *P*-values for the comparison between HCs and patients with TAVS. Boxplots further illustrate the results for the significant comparisons. Upon *ex vivo* stimulation with PI:C, ≥30% of the participants did not produce TNFα. Therefore, this condition was analysed as a categorical variable (reaction/no reaction). HC *n* = 63, S-TAVS *n* = 69, M/M-TAVS *n* = 48. S-TAVS: IL-1β after PolyI:C and LPS + MSU stimulation *n* = 68. M/M-TAVS IL-1β after LPS + MSU stimulation *n* = 47. (*B*–*D*) HCs are compared with patients with mild/moderate TAVS and patients with severe TAVS, respectively. Variables are compared with linear regression or logistic regression and are corrected for age, sex, BMI, smoking, SBP, glucose, LDL-cholesterol, and season and year of blood drawing applying manual backward covariate selection. Correction for multiple testing was performed using the Benjamini–Hochberg method with a 10% false discovery rate. (*C* and *D*) *P*-values are given in black if they remained significant after correction for multiple testing and in grey if they did not remain significant. In the boxplot, the median value is defined by the in-box line, the 25th and 75th percentiles by the hinges, and the range of the results by the whiskers. *P ≤ 0.05, **P ≤ 0.01, ***P ≤ 0.001, ****P ≤ 0.0001.

Patients with S-TAVS had higher leukocyte counts and patients with M/M and S-TAVS had higher lymphocytes, monocytes, and eosinophils (Sysmex XN-450 automated haematology analyser; *Figure 1B*). TAVS patients had higher circulating interleukin-6 (IL-6) [2.31 (1.85–3.09) pg/mL in M/M-TAVS; 3.17 (1.98–4.85) in S-TAVS, 1.27 (0.92–1.82) in HC, *P* < 0.001]. There was no difference in C-reactive protein.

We performed flow cytometry on fresh whole blood, using mouse monoclonal antibodies against cluster of differentiation (CD) 3, CD11b, CD11c, CD14, CD16, CD19, CD41, CD45, CD56, C-C chemokine receptor (CCR) 2, CCR5, and HLA-DR (CytoFlex cytometer). There were no differences in the percentage of CD14^++^CD16^−^ classical monocytes, CD14^++^CD16^+^ intermediate monocytes, and CD14^+^CD16^++^ non-classical monocytes. S-TAVS patients had a higher percentage of CCR2^+^ cells compared to HC in the intermediate [84.6% (77.4–89.1) vs. 74.0% (64.0–81.0), *P* < 0.001] and non-classical monocytes [5.2% (3.1–11.0) vs. 2.6% (1.6–5.0), *P* = 0.003]. CCR2 expression [median fluorescence intensity (MFI)] was significantly higher in total, classical, and intermediate monocytes in M/M- and S-TAVC patients (*Figure 1C*).

In contrast, HLA-DR expression was lower in TAVS compared to HC (*Figure 1C*). There was no difference in CCR5, CD11b, CD11c, and CD41 expressions. There was a positive correlation between IL-6 and CCR2 MFI (Spearman *r* = 0.2386, *P* = 0.001) and a negative correlation between IL-6 and HLA-DR MFI (Spearman *r* = −0.2782, *P* < 0.001).

To assess monocyte function, we stimulated freshly isolated peripheral blood mononuclear cells (PBMCs) for 24 h with RPMI (negative control), 10 ng/mL *E. coli* lipopolysaccharide (LPS), 10 μg/mL Pam3Cys (P3C), 1 × 106/mL heat killed *Candida albicans conidia* (*C. albicans*; UC 820, in house), 30 μg/mL polyinosinic:polycytidylic acid (PolyI:C), and 300 μg/mL monosodium urate crystals (MSU) combined with 10 ng/mL LPS. We stimulated PBMC instead of purified monocytes to preserve the cell–cell interactions known to be important *in vivo* and thus use a system that is more relevant to real-life situations. We measured IL-1β, IL-6, tumour necrosis factor (TNF)-α, IL-1Ra and IL-10 in the supernatants with DuoSet ELISA kits. PBMCs from patients with S-TAVS showed higher production of IL-1β, and TNFα after PolyI:C stimulation; other differences were not statistically significant (*Figure 1D*). After correction for multiple testing, no differences were observed between patients with M/M-TAVS and HCs (*Figure 1D*).

To assess immune parameters independent of concomitant atherosclerotic cardiovascular disease (ASCVD), all analyses were repeated after excluding patients with a history of cardiovascular events and/or a coronary stenosis ≥ 30%. In the 19 patients with M/M-TAVS and 27 patients with S-TAVS who did not have concomitant ASCVD, we confirmed higher CCR2 expression in total, classical, and intermediate monocytes and lower HLA-DR expression in all subsets of monocytes. The analysis of PBMC cytokine production capacity confirmed higher production of TNFα and IL-1β after PolyI:C stimulation in S-TAVS patients, but significance was lost after multiple testing correction. Additionally, the results for these parameters also remain significant after correction for statin use (data not shown).

The chemokine receptor CCR2 mediates monocyte egress from the bone marrow and monocyte recruitment to sites of inflammation and strongly contributes to atherogenesis.^5^ In individuals with cardiovascular risk, monocyte CCR2 expression is positively correlated with arterial wall inflammation.^6^ Studies on the involvement of CCR2 in CAVD are scarce. The CCR2 ligand CCL-2 is expressed in calcified aortic valves and increased by inflammatory stimuli.^7^ During CAVD progression, there is an increased macrophage number in human valves.^8^ We propose that the higher monocyte CCR2 expression facilitates monocyte recruitment to the aortic valve and contributes to valvular inflammation.

Plasma IL-6 concentrations were higher in TAVS patients, positively correlated with monocyte CCR2 expression and negatively with HLA-DR expression (monocyte bioactivity). IL-6 is produced by several cells, including monocytes, macrophages, fibroblasts, and endothelial cells and is an important cytokine in CAVD pathology as it promotes valvular inflammation and calcification.^9^ Aortic valves of patients with AVS show increased IL-6 RNA expression,^9^ and certain polymorphisms of the *IL-6* gene are associated with the development of CAVD.^10^ A lower HLA-DR expression indicates immunosuppression, which is observed in conditions of acute systemic inflammation, such as in sepsis.^11^ In our study, TAVS patients had low-grade systemic inflammation with higher IL-6 plasma levels, and it is likely that this contributed to the lower HLA-DR expression. Indeed, IL-6 can downregulate HLA-DR expression.^12^ In addition, the lower HLA-DR expression might also be driven by TGF-β signalling. TGF-β is able to reduce monocyte HLA-DR expression,^13^ and TGF-β plasma concentrations are higher in older patients with AVS.^14^ Other factors that can affect HLA-DR expression in human monocytes are TNFα, TLR2, and TLR4 agonists (upregulation) and IL-10 (downregulation).^15^ More research is needed to elucidate the regulation of HLA-DR in TAVS.

Lastly, we assessed monocyte function and demonstrated higher PBMC cytokine production capacity in TAVS patients. This finding resembles previous findings in patients with ASCVD.^2^ The higher cytokine production was most pronounced in response to PolyI:C, which interacts with Toll-like receptor 3 (TLR3). TLR3 is a lysosomal pattern-recognition receptor that binds double-stranded RNA and promotes pro-inflammatory responses. Double-stranded RNA can be released from damaged/stressed cells in diseased aortic valves, and endogenous TLR3 activation can contribute to experimental aortic valve stenosis.^16^ These findings underscore the potential significance of our finding that in patient with TAVS, circulating monocytes are primed to a hyperresponsiveness to TLR3 stimulation.

In summary, we revealed that TAVS patients have higher systemic inflammation than HCs, with higher circulating leukocytes and IL-6, and have an altered monocytes phenotype and function, characterized by higher CCR2 expression, lower HLA-DR expression, and a higher PBMC cytokine production capacity, mainly after stimulation with PolyI:C. These parameters could potentially be used as new biomarkers of CAVD progression, or new targets for pharmacological treatment strategies. Further validation in prospective longitudinal studies is essential.

## Data Availability

The data underlying this article will be shared on reasonable request to the corresponding author.
